# Bu-Zhong-Yi-Qi Decoction, the Water Extract of Chinese Traditional Herbal Medicine, Enhances Cisplatin Cytotoxicity in A549/DDP Cells through Induction of Apoptosis and Autophagy

**DOI:** 10.1155/2017/3692797

**Published:** 2017-01-05

**Authors:** Ning Yu, Ying Xiong, Chun Wang

**Affiliations:** ^1^Key Laboratory of Ministry of Education for TCM Viscera-State Theory and Applications, Liaoning University of Traditional Chinese Medicine, 79 Chongshan Eastern Road, Huanggu District, Shenyang 110847, China; ^2^The First Clinical College, Liaoning University of Traditional Chinese Medicine, 79 Chongshan Eastern Road, Huanggu District, Shenyang 110847, China; ^3^Department of Cell Biology, Basic Medical College, Liaoning University of Traditional Chinese Medicine, 79 Chongshan Eastern Road, Huanggu District, Shenyang 110847, China

## Abstract

Cisplatin is one of the most active cytotoxic agents for non-small cell lung cancer (NSCLC) treatment. However, the development of cisplatin resistance is common. Bu-Zhong-Yi-Qi decoction (BZYQD), a Chinese traditional herbal medicine, is widely used for the enhancement of antitumor effect in other medications. In this study, we evaluated the effect and drug-resistance reversal mechanism of BZYQD combined with cisplatin on cisplatin-resistant A549/DDP cells. Our results showed that BZYQD exhibited direct cytotoxic and chemosensitizing effects. Cotreatment with BZYQD and cisplatin induced intrinsic apoptotic pathways which were measured by condensed nuclear chromatin, Annexin V/PI apoptosis assay, and apoptosis related proteins expression. In addition, cotreatment with BZYQD and cisplatin also activated autophagy, as indicated by an increase in LC3 puncta, classical autophagosomes and/or autolysosomes, and an accumulation of LC3-II and ATG7 protein. Finally, cotreatment with BZYQD and cisplatin resulted in the generation of ROS and scavenging ROS by NAC almost completely suppressing cell death. These results suggest that cotreatment with BZYQD and cisplatin might reverse cisplatin resistance by inducing ROS accumulation, which activates apoptosis and autophagy by oxidative stress. The combination of BZYQD and cisplatin may represent a novel approach in treatment for NSCLC and thus offer a new target for chemotherapy.

## 1. Introduction

Lung cancer is the most common cause of cancer-related death worldwide, with non-small cell lung cancer (NSCLC) accounting for approximately 80*～*85% of all lung cancers [1, 2]. Cisplatin-based combination chemotherapy is currently the standard drug treatment for NSCLC. However, as a result of its frequent usage, resistance to cisplatin has become more common and has been recognized as a reason for the failure of many cancer therapies [3].

Classical traditional Chinese medicine (TCM), which includes acupuncture, traumatology, manipulative therapies, and Chinese herbal products, has been an important part of health care in China for hundreds of years. Bu-Zhong-Yi-Qi decoction (BZYQD), also called Bu-Zhong-Yi-Qi-Tang in China and Bojungikki-tang or Hochu-ekki-to in Japan, comprises crude ingredients that are extracted from eight herbs (Table 1). It is a well-known formula in traditional Chinese medicine identified as an effective medication to improve the quality of life and nutritional status of patients [4–7]. BZYQD is useful, not only for the improvement of daily activity of chronic fatigue syndrome, but also for the enhancement of antitumor effects of other drugs [8–10]. BZYQD has been reported to induce gastric cancer cell death by nonapoptotic mechanisms and to induce human ovarian cancer cell death by apoptotic mechanisms [11, 12]. Moreover, we have confirmed that BZYQD can reduce gastrointestinal injury by suppressing inflammatory cytokine upregulation and reduce renal injury in mice by an antioxidant mechanism in cancer chemotherapy [13, 14]. But whether or not BZYQD can act as a resistance reversal agent in cancer chemotherapy to increase chemotherapy sensitivity is still unknown.

In the present study, we evaluated the effect of BZYQD in combination with cisplatin on human non-small cell lung cancer cells and also its possible drug-resistance reversal mechanism.

## 2. Materials and Methods

This work was supported by the National Natural Science Foundation of China (81573856); the Science and Technology Foundation of Liaoning Province (2015020379); and the Open Fund of Key Laboratory of Ministry of Education for TCM Viscera-State Theory and Applications, Liaoning University of Traditional Chinese Medicine (zyzx1603).

### 2.1. Preparation and Administration of BZYQD

Eight herbs (Table 1) were put into a 20-fold volume of distilled water, decocted from 80°C to 100°C, filtered, and concentrated at 40°C to 80°C. The extract was spray-dried in a hot air stream. The powder forms of BZYQD were dissolved in RPMI 1640 medium up to 5000 *μ*g/ml, vortexed at room temperature for 1 minute, and then incubated under rotation at 37°C for 1 hour. These solutions were centrifuged at 5000 rpm for 5 minutes to remove any insoluble ingredients. The supernatants were passed through a 0.22 *μ*m filter for sterilization [4, 15]. The quality of the BZYQD is controlled by measuring the contents with high-performance liquid chromatography (HPLC) [14].

### 2.2. Cell Culture

Human lung carcinoma A549/DDP cells (cisplatin-resistant cells) were obtained from the cancer hospital at the Chinese Academy of Medical Sciences (Beijing, China) and cultured in RPMI 1640 containing 10% fetal bovine (Hyclone, Cramlington, UK) and 1% penicillin and streptomycin (Gibco, Carlsbad, CA, USA) in a humid atmosphere of 5% CO_2_/95% air. The A549/DDP cells medium additionally contained 2 *μ*g/ml cisplatin to maintain the cells' drug-resistant phenotype [1]. Cells in the logarithmic phase of growth were used for all experiments.

### 2.3. Cell Viability and Growth Inhibition Assay

The cell viability and growth inhibition of BZYQD on A549/DDP cells were determined by counting viable cells with a Cell Counting Kit-8 (Vazyme, Nanjing, China). An equal number of cells of the six cell lines (5 × 10^3^ cells/well) in 100 *μ*l of conditioned medium were seeded into a 96-well microplate and incubated overnight. The cells were then treated with various concentrations of BZYQD (0, 50, 100, 250, 500, 1000, 2500, and 5000 *μ*g/ml). After incubation for 26 hours, 10 *μ*l of Cell Counting Kit-8 solution was added to each well, and the plates were further incubated for 4 hours at 37°C. For the assay of the synergistic effect of BZYQD on cisplatin, A549/DDP cells were pretreated with PBS as control and various BZYQD concentrations (100, 250, and 500 *μ*g/ml, the approximate IC_5_, IC_10_, and IC_20_ of BZYQD drug exposure concentrations) for 2 hours before any other medium was added. Then cisplatin (0, 20, 50, 100, 200, and 500 *μ*g/ml) was added to A549/DDP cells for another 24 hours of incubation before assaying for cell viability. To confirm the mechanism of growth inhibition induced by coincubation with BZYQD and cisplatin, A549/DDP cells were pretreated with caspase inhibitor z-Val-Ala-Asp-fluoromethylketone (z-VAD-fmk, 20 *μ*M) for 2 hours, autophagy inhibitor 3-Methyladenine (3-MA, 10 mM) for 4 hours, or reactive oxygen species (ROS) scavenger N-acetyl-cysteine (NAC, 2.5 mM) for 3 hours. Then, BZYQD and cisplatin were similarly processed. Subsequently, cell viability was evaluated by Cell Counting Kit-8 assay. The absorbance was measured at 460 nm. Dose-response curves were plotted on a semilog scale as the percentage of the control cell number, which was obtained from the sample with no drug exposure.

### 2.4. Hoechst Staining

A549/DDP cells were seeded on coverslips on a 6-well plate and initially pretreated with BZYQD (100, 250, and 500 *μ*g/ml) for 2 hours. Then 40 *μ*g/ml cisplatin (about the concentration of IC_20_) was added to A549/DDP cells for another 24 hours of incubation. The attached cells were washed with PBS and fixed in 70% ethanol for 30 minutes and then incubated with 10 mg/ml bisbenzimide trihydrochloride (Hoechst 33258) staining solution (Beyotime, China) for 30 minutes. After treatment, cells were washed with PBS and then observed under a fluorescence microscope (Leica, Germany).

### 2.5. Annexin V/PI Staining Assay

To confirm the apoptosis induced by coincubation with BZYQD and cisplatin, the percentage of apoptotic cells was measured by dual staining of cells with Annexin V and propidium iodide (PI). A549/DDP cells were seeded in 6-well plates and treated with BZYQD and cisplatin according to the aforementioned method. The cells were then collected and treated as outlined in the protocol of the Annexin V-fluorescein isothiocyanate (FITC) apoptosis detection kit (SAB, Pearland, Texas, USA). The percentages of apoptotic cells were determined and analyzed by a flow cytometer (BD, Franklin Lakes, New Jersey, USA).

### 2.6. Evaluation of LC3 Fluorescent Puncta

Cells grown on glass coverslips were fixed in 2% formaldehyde for 10 minutes, permeabilized with 0.5% Triton X-100 in PBS for 30 minutes, and treated with 2% BSA in PBS for 1 hour, at room temperature. Next, samples were incubated with microtubule-associated protein light chain-3-II (LC3-II) antibody (1 : 100, Proteintech, Chicago, USA) overnight at 4°C, rinsed three times with 0.01% Triton X-100 in PBS, and incubated for 30 minutes with a secondary antibody (FITC-conjugated rabbit IgG at 1 : 200, ZSGB BIO, China) at 37°C. Afterwards, cells were further stained with DAPI and observed under a fluorescence microscope (Leica, Germany) [16].

### 2.7. Transmission Electron Microscopy

Cells were then fixed for 2 hours with 2.5% glutaraldehyde in 0.1 M phosphate buffer (pH 7.4), postfixed in 1% OsO_4_ dehydrated in graded ethanol, and then embedded in epoxy resin. Ultra sections were observed under a transmission electron microscope (model RILI H-7500; Hitachi, Japan) at 80 kV [17].

### 2.8. Determination of Intracellular Reactive Oxygen Species

The generation of ROS production in A549/DDP cells was monitored with an intracellular oxidative stress ROS assay kit (GENMED, Shanghai, China) [17]. After cotreatment with BZYQD and cisplatin, cells were washed with PBS and then loaded with dichlorodihydrofluorescein diacetate (DCFH-DA) for 30 minutes at 37°C as per protocol. Subsequently, cells were removed, washed, and resuspended in PBS for analysis by DCF fluorescence. Next, the 2′-7′-dichlorofluorescein (DCF) fluorescence stained cells were measured by FCM and analyzed on Cell Quest software flow cytometer (BD, New Jersey, USA).

### 2.9. Western Blot Analysis

After treatment, A549/DDP cells were lysed and subjected to western blot analysis as described previously [18]. The blots were probed with antibodies against glyceraldehyde 3-phosphate dehydrogenase (GAPDH; SAB, USA), cleaved caspase 3 (SAB, USA), cleaved poly (adenosine diphosphate-ribose) polymerase (PARP; SAB, USA), Bax (CST, USA), Bcl–2 (CST, USA), and LC 3-II and Atg7 (BIOSYNTHESIS, Beijing, China). Horseradish peroxidase-conjugated secondary antibodies (ZSGB BIO, Beijing, China) were used in conjunction with an enhanced chemiluminescence detection system (GE Amersham, USA). Staining was quantified by scanning densitometry.

### 2.10. Statistical Analyses

Each experiment was performed separately at least three times. The data are expressed as the mean ± SD. A statistical comparison between different groups was performed by an ANOVA (analysis of variance) test. *P* < 0.05 indicates significance, and NS indicates no significant difference (*P* > 0.05). Statistical analyses were conducted using SPSS 15.0.

## 3. Results

### 3.1. Direct Cytotoxic Effect of BZYQD on A549/DDP Cells

**Figure 1:**
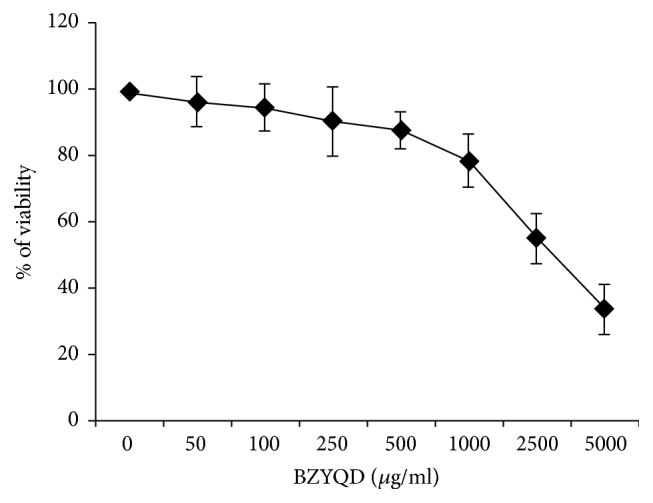
Direct cytotoxic effect of BZYQD on A549/DDP cells. A549/DDP cells were treated with various concentrations (0, 50, 100, 250, 500, 1000, 2500, and 5000 *μ*g/ml) of BZYQD for 24 h. The cell viability was determined by the Cell Counting Kit as described in the text. Each data point represents the mean ± SD of results from four individual measurements. BZYQD: Bu-Zhong-Yi-Qi decoction.

We first examined the direct effect of BZYQD on the growth of A549/DDP cells in vitro. The viability of the treated cell lines was determined as the ratio between viable treated cells and viable untreated control cells. As shown in the Figure 1, BZYQD displays direct antitumor effects. The IC_50_ were 3890 *μ*g/ml; and IC_5_, IC_10_ and IC_20_ were 104, 236 and 486 *μ*g/ml, respectively.

### 3.2. Combination of BZYQD and Cisplatin on Induction Cytotoxicity

**Figure 2:**
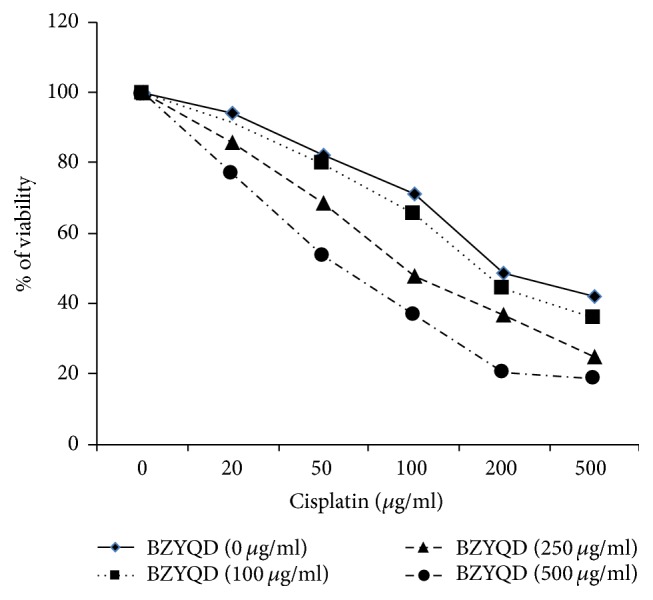
Effects of BZYQD on the cytotoxicity induced by cisplatin. A549/DDP cells were initially pretreated with 100, 250, and 500 *μ*g/ml BZYQD (the approximate IC_5_, IC_10_, and IC_20_ of drug exposure concentrations) for 2 h. Then cisplatin (0, 20, 50, 100, 200, and 500 *μ*g/ml) was added for another 24 h. The cell viability was determined by the Cell Counting Kit. Each data point represents the mean ± SD of results from four individual measurements. BZYQD: Bu-Zhong-Yi-Qi decoction.

BZYQD exhibits a pronounced effect on the enhancement of cisplatin-induced cytotoxicity (Figure 2), with IC_50_ values of cisplatin ranging from 241.8 to 223.5, and 123.1 and 97.7 *μ*g/ml after coexposure with BZYQD 100, 250, and 500 *μ*g/ml, respectively.

### 3.3. Combination of BZYQD and Cisplatin on Induction Cells Apoptosis

**Figure 3:**
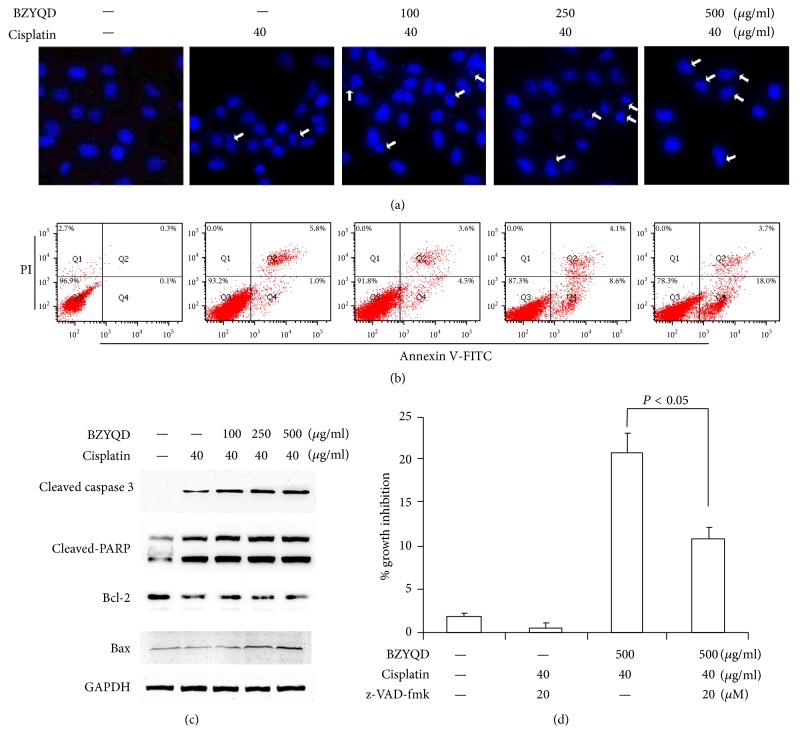
Combination treatment with BZYQD and cisplatin leads to apoptosis induction in A549/DDP cells. (a) A549/DDP cells were pretreated with 100, 250, and 500 *μ*g/ml BZYQD for 2 h, and then 40 *μ*g/ml cisplatin was added for another 24 h. Photomicrographs of representative fields of cells stained with Hoescht-33258 to evaluate nuclear chromatin condensation. (b) The percentage of AnnexinV+/PI− (early apoptotic cells, lower right), AnnexinV+/PI+ (late apoptotic cells, upper right), AnnexinV−/PI− (viable cells, lower left), and AnnexinV−/PI+ (necrotic cells, upper left) cells is shown. (c) Cell lysates were prepared and subjected to immunoblotting with antibodies to cleaved caspase 3, cleaved-PARP, Bcl-2, Bax, and GAPDH. (d) A549/DDP cells were pretreated with 20 *μ*M z-VAD-fmk 2 h prior to cotreatment with BZYQD and cisplatin. After incubation, cell growth inhibition was determined by the Cell Counting Kit as described previously. The results are expressed as the mean ± SD of four independent experiments. (ANOVA followed by Bonferroni's test.) BZYQD: Bu-Zhong-Yi-Qi decoction; PI: propidium iodide; GAPDH: glyceraldehyde 3-phosphate dehydrogenase; z-VAD-fmk: z-Val-Ala-Asp-fluoromethylketone.

We next assessed whether or not the enhanced cytotoxicity to cisplatin by BZYQD was due to the induction of apoptosis. Apoptosis was evaluated by noting morphological changes of condensed nuclear chromatin. A549/DDP cells exposed to cisplatin (40 *μ*g/ml) combined with various concentrations of BZYQD show an increase in dose-dependent apoptosis when compared to PBS and cisplatin alone (Figure 3(a)). Using Annexin V/PI apoptosis detection by FCM as another independent assay for apoptosis measurement, we confirmed the findings from morphologic fluorescent microscopy (Figure 3(b)). Finally, we analyzed the activation of caspase 3 characteristics for the induction of apoptosis as well as the inactivation of PARP, a DNA repair factor, by immunoblotting. Caspase 3 activation and PARP inactivation/cleavage increased gradually following cotreatment with increasing BZYQD and cisplatin (Figure 3(c)). Interestingly, the protein expression of antiapoptotic protein Bcl-2 and proapoptotic protein Bax was detected, as well. Figure 3(c) shows that cotreatment with BZYQD and cisplatin significantly reduced protein expressions of Bcl-2 and increased the protein levels of Bax.

To confirm the cell apoptosis induced by cotreatment with BZYQD and cisplatin, A549/DDP cells were pretreated with caspase inhibitor z-VAD-fmk, followed by cotreatment with high-dosage BZYQD (500 *μ*g/ml) and cisplatin. We found that z-VAD-fmk partially abolishes cell growth inhibition induced by cotreatment with BZYQD and cisplatin (Figure 3(d)), which suggests that cotreatment with BZYQD and cisplatin plays an important role in restoring cisplatin sensitivity in cisplatin-resistant NSCLC cells.

### 3.4. Combination of BZYQD and Cisplatin Triggered Cells Autophagy

**Figure 4:**
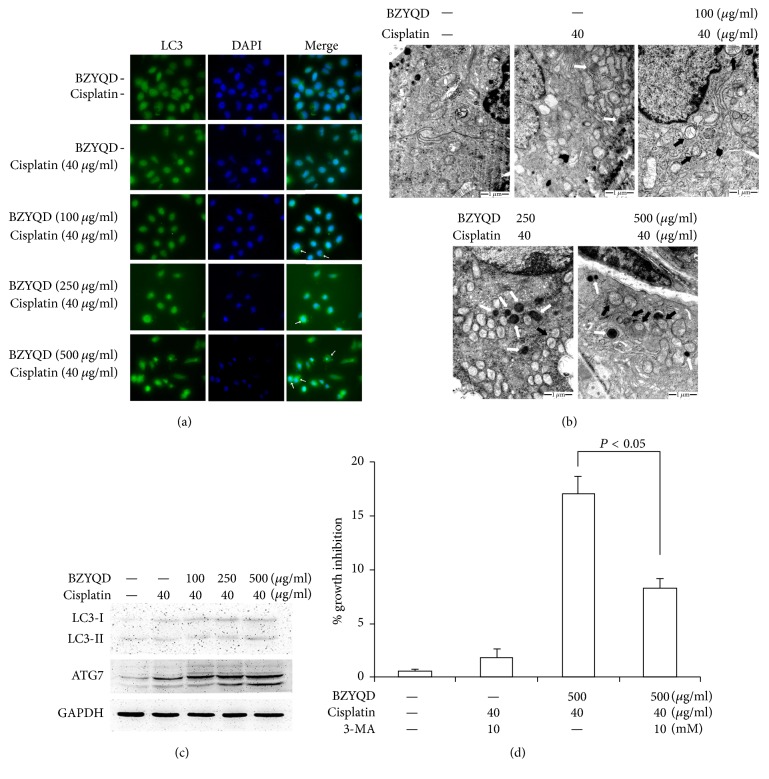
Combination treatment with BZYQD and cisplatin leads to autophagy induction in A549/DDP cells. (a) A549/DDP cells were pretreated with 100, 250, and 500 *μ*g/ml BZYQD for 2 h, and then 40 *μ*g/ml cisplatin was added for another 24 h. After drug cotreatment, cells were stained by indirect immunofluorescence to visualize LC3 puncta (green) by fluorescence microscopy. Cells were then stained with DAPI to visualize the nuclei (blue). (b) The formation of autophagosomes in treated cells was evaluated by TEM. Control cells seemed to be normal in both amount and ultrastructure morphology of mitochondria and lysosomes. Solo-cisplatin treated cells showed increasing numbers, swelling, and vacuolated mitochondria. BZYQD and cisplatin cotreated cells showed increasing the number of autophagosomes and secondary lysosomes, as well as autophagosomes formation. “Black arrow” indicates autophagosomes and “white arrow” indicates secondary lysosomes. (c) LC3-II and ATG7 were examined by western blot. GAPDH was a loading control. (d) A549/DDP cells were pretreated with 10 mM 3-MA for 4 h prior to cotreatment with BZYQD and cisplatin. After incubation, cell growth inhibition was determined by the Cell Counting Kit as described previously. Results are expressed as the mean ± SD of four independent experiments. (ANOVA followed by Bonferroni's test.) BZYQD: Bu-Zhong-Yi-Qi decoction; LC3: microtubule-associated protein light chain-3; GAPDH: glyceraldehyde 3-phosphate dehydrogenase; 3-MA: 3-methyladenine.

Previous reports have indicated that causing cisplatin resistance in lung carcinoma cells after long-term drug exposure results in autophagy [19]. We investigated autophagy as a possible alternative mode of cell death activated by exposure to combined BZYQD and cisplatin. Autophagy induction was first confirmed by analysis of microtubules associated with LC3 redistribution, which changes its form from LC3-I to LC3-II and recruits to the autophagosome membrane [20]. As shown in Figure 4(a), diffuse cytoplasmic distribution of green fluorescence was observed in all groups. However, an increase in the characteristic redistribution of LC3 punctate vesicular structures was observed in cells exposed to BZYQD and cisplatin and, to a much greater extent, in cells exposed to high concentrations of BZYQD and cisplatin. Subsequently, classical autophagosomes and/or autolysosomes were observed by transmission electron microscopy in all drug-treated cells and were especially common in those cells cotreated with BZYQD and cisplatin (Figure 4(b)). Autophagy-specific markers LC3-II and ATG7 were also used to examine the autophagic levels in this process by immunoblot analysis. As shown in Figure 4(c), LC3-II and ATG7 were both upregulated following BZYQD and cisplatin cotreatment. We also pretreated A549/DDP cells with 3-MA, a well-known inhibitor of autophagosomal lysosome formation, and found an abolished cell growth inhibition induced by cotreatment with high-dosage BZYQD and cisplatin (Figure 4(d)).

### 3.5. Combination of BZYQD and Cisplatin-Induced ROS Generation in A549/DDP Cells

**Figure 5:**
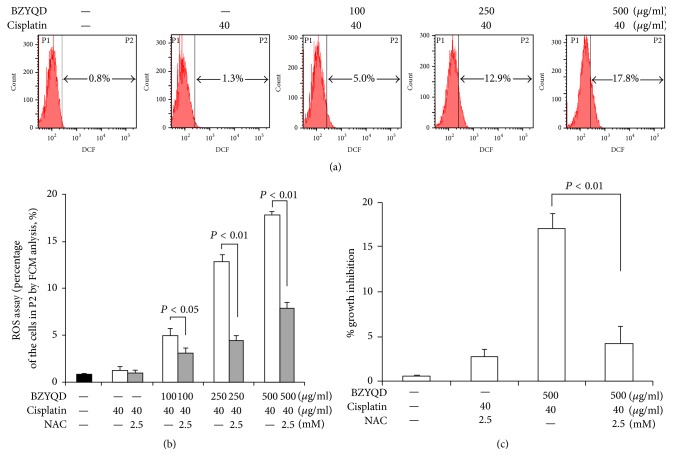
ROS generation led to apoptosis and autophagy in BZYQD and cisplatin cotreated A549/DDP cells. (a) A549/DDP cells were pretreated with 100, 250, and 500 *μ*g/ml BZYQD for 2 h, and then 40 *μ*g/ml cisplatin was added for another 24 h. After drug cotreatment, cells were labeled with DCFH-DA and the fluorescent intensity of the oxidized product DCF in individual cells was detected by flow cytometry. (b) Statistical analysis of the cell numbers in area P2 in (a). The results are expressed as the mean ± SD of four independent experiments. (ANOVA followed by Bonferroni's test.) (c) A549/DDP cells were pretreated with 2.5 mM NAC 3 h prior to cotreatment with BZYQD and cisplatin. After incubation, cell growth inhibition was determined by the Cell Counting Kit as described previously. The results are expressed as the mean ± SD of four independent experiments. (ANOVA followed by Bonferroni's test.) ROS: reactive oxygen species; BZYQD: Bu-Zhong-Yi-Qi decoction; DCFH-DA: dichlorodihydrofluorescein diacetate; DCF: 20,70-dichlorofluorescein; GAPDH: glyceraldehyde 3-phosphate dehydrogenase; NAC: N-acetylcysteine.

We analyzed the intracellular ROS production, which functions as an initial mediator inflicting damage on intracellular components such as DNA, proteins, and amino acids [21]. As shown in Figure 5(a), a dose-dependent increase of ROS levels was detected in A549/DDP cells with the cotreatment of BZYQD and cisplatin. However, pretreatment with NAC, a well-known antioxidant, for 3 hours effectively inhibited ROS production (Figure 5(b)) and inhibited A549/DDP cell growth significantly when cotreated with high-dosage BZYQD and cisplatin (Figure 5(c)). Therefore, ROS did contribute towards the induced demise of A549/DDP cells when cotreated with BZYQD and cisplatin.

## 4. Discussion

Cisplatin-based chemotherapy is widely used in the treatment of advanced lung cancer with an approximate survival rate of 5 years. However, despite effective cytotoxicity of cisplatin in initial treatment, cancer cells frequently develop resistance to cisplatin as a result of repeated use [22]. Cisplatin resistance is common, and the mechanisms of cisplatin resistance appear to be multifactorial. Specifically, apoptosis-resistance results from the altered cell death pathways, and autophagy induction functions as a cytoprotective mechanism in the chemoresistance of cancer cells [2, 22]. Therefore, the development of novel agents that target apoptosis and autophagy is important for improving the survival rate of lung cancer patients.

Apoptosis is a mechanism for programmed cell death that is regulated by cellular signaling pathways. The dysregulation of Bcl-2 family proteins, which includes overexpression of antiapoptotic members and decreased expression of proapoptotic members, is required for cancer development, contributes to apoptotic resistance, and is associated with chemoresistance, as well [22, 23]. In this report, we investigated the mechanisms of BZYQD, a Chinese herbal compound, for reversing cisplatin resistance in NSCLC cells. Our results showed that BZYQD displays a growth inhibitory effect on cisplatin-resistant lung cancer cells (A549/DDP), which confirms findings from a previous study investigating the direct antitumor effects of BZYQD in cancer cell lines, like ovarian cancer cells [12]. Furthermore, BZYQD exhibits a pronounced effect on the enhancement of cisplatin-induced cytotoxicity by decreasing cisplatin IC_50_ from 241.8 to 97.7 *μ*g/ml when coexposed to 500 *μ*g/ml BZYQD. In addition, we also found that the growth inhibition induced by BZYQD and cisplatin cotreatment in A549/DDP cells occurs through apoptosis, as confirmed by Annexin V/PI apoptosis detection and characteristic apoptosis protein expression (caspase 3 activation and PARP inactivation/cleavage). BZYQD and cisplatin cotreatment also upregulated Bax and downregulated Bcl-2 expressions, which connects with apoptotic resistance and chemoresistance from the intrinsic mitochondrial pathway. The pan-caspase inhibitor, z-VAD-fmk, also partially blocks the BZYQD and cisplatin-induced apoptosis, suggesting that the cotreatment occurs through a caspase-dependent pathway. However, the incomplete inhibition of apoptosis by z-VAD-fmk suggests that the operation of an additional mechanism is also involved.

Autophagy is a type of programmed cell death that occurs in both physiological and pathophysiological environments. Autophagy plays a paradoxical role in cancer cell survival and death, as it is the removal process of aging or damaged organelles that helps maintain cellular homeostasis and helps cell survival during tumor progression [24]. However, excessive autophagy or improper activation can lead to apoptosis and autophagic cell death which also promotes cancer cell death [25]. Furthermore, autophagy plays an equally complicated role in the mechanisms of cancer cell chemoresistance. Autophagy is recognized as a cytoprotective process, as a low dose of cisplatin exposure induces chemoresistance originating from an autophagy initiation [24]; however, the idea of autophagic reduction caused by long-term exposure to cisplatin has also been previously considered [19]. In chemotherapy, inducing autophagic cell death is an efficient way to induce cell death, specifically for apoptosis-resistant cells [25]. Some studies have demonstrated that when cisplatin is unable to trigger an apoptotic response in those apoptosis-resistant cells, autophagy, as a mechanism of cytotoxicity, can promote cell death [26]. In our study, we confirmed that cytotoxic autophagy is activated following BZYQD and cisplatin cotreatment in A549/DDP cells by the formation of LC3 puncta and autophagosomes, as well as by the accumulation of the proteins LC3-II and ATG7. It has been reported that BZYQD showed the most pronounced effect in augmenting mitomycin C-induced cytotoxicity in human gastric cancer cells, but the cytotoxic effect is indicated in nonapoptotic mechanisms [9]. Therefore, we hypothesize that this nonapoptotic mechanism might be autophagy induced by BZYQD and mitomycin C cotreatment in gastric cancer cells, as well.

So far, our study has shown that the mechanism of cell death induced by combined treatment with BZYQD and cisplatin involves both apoptosis and autophagy for reversing cisplatin resistance. In addition, it provides an interesting link between these two pathways and the loss of cell viability. Accumulating evidence reveals that autophagy and apoptosis can cooperate, antagonize, or assist each other, thus influencing cell fate [20]. In our study, the use of the pan-caspase inhibitor z-VAD-fmk or autophagy inhibitor 3-MA only partially affected the cell viability induced by cotreatment with BZYQD and cisplatin on A549/DDP cells. These results indicate that, in this specific context, apoptosis and autophagy are collectively committed to BZYQD's cisplatin sensitivity in cisplatin-resistance cells. Analysis of the relationship between apoptosis and autophagy demonstrates that ROS generation contributes to the process of these two programmed cell death mechanisms [27]. Our data show that cotreatment with BZYQD and cisplatin induces ROS generation in cisplatin-resistance A549/DDP cells and that scavenging ROS by NAC almost completely suppresses cell death inhibition.

In conclusion, this study is the first report to delineate the mechanistic pathways for the apoptosis and autophagy for reversing chemoresistance NSCLC cells that is induced by cotreatment with BZYQD and cisplatin. This combined treatment increases the intracellular ROS levels, causing the onset of apoptosis and activating an autophagy program by passively allowing oxidative stress to accumulate. These findings should highlight the future of BZYQD, an important classical TCM, as a potential chemotherapy sensitizer in the treatment of different human cancers.

## Figures and Tables

**Table 1:** Herbal constituents of BZYQD.

	Pharmaceutical name	Part used	Amount (g)
(1)	*Astragalus membranaceus *(Fisch.) Bge. var. *mongholicus* (Bge.) Hsiao	Root	18
(2)	*Glycyrrhiza uralensis *Fisch.	Root and rhizome	9
(3)	*Codonopsis pilosula *(Fisch.) Nannf.	Root	6
(4)	*Angelica sinensis *(Oliv.) Diels	Root	3
(5)	*Citrus reticulate *Blanco	Pericarp	6
(6)	*Cimicifuga heracleifolia *Kom.	Rhizome	6
(7)	*Bupleurum chinense *DC.	Root	6
(8)	*Atractylodes macrocephala *Koidz.	Rhizome	9

	Total		63

BZYQD: Bu-Zhong-Yi-Qi decoction.
